# Supplementary figures and images for: Improving PD-1 blockade plus chemotherapy for complete remission of lung cancer by nanoPDLIM2
Source: eLife. 2024 Dec 24;12:RP89638. doi: 10.7554/eLife.89638 (PMC11668523; doi:10.7554/eLife.89638)

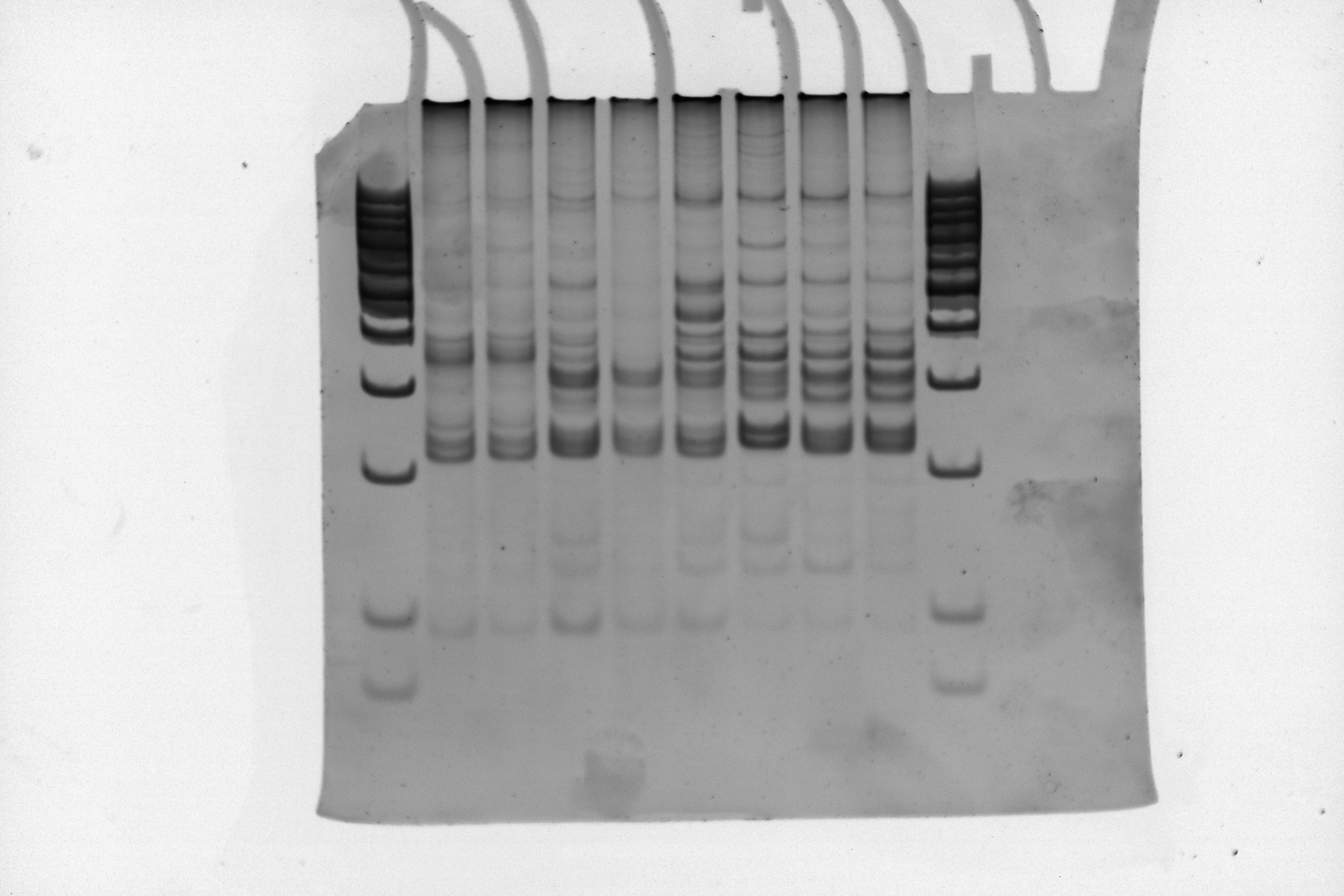

Supplement: Figure 1—source data 2. [file elife-89638-fig1-data2.zip › Figure1SourceData2_Fig1E_DNAGelsOriginal/20150912 patients-2488-2656-2411-2427 D8S1786-6-invert.tif]

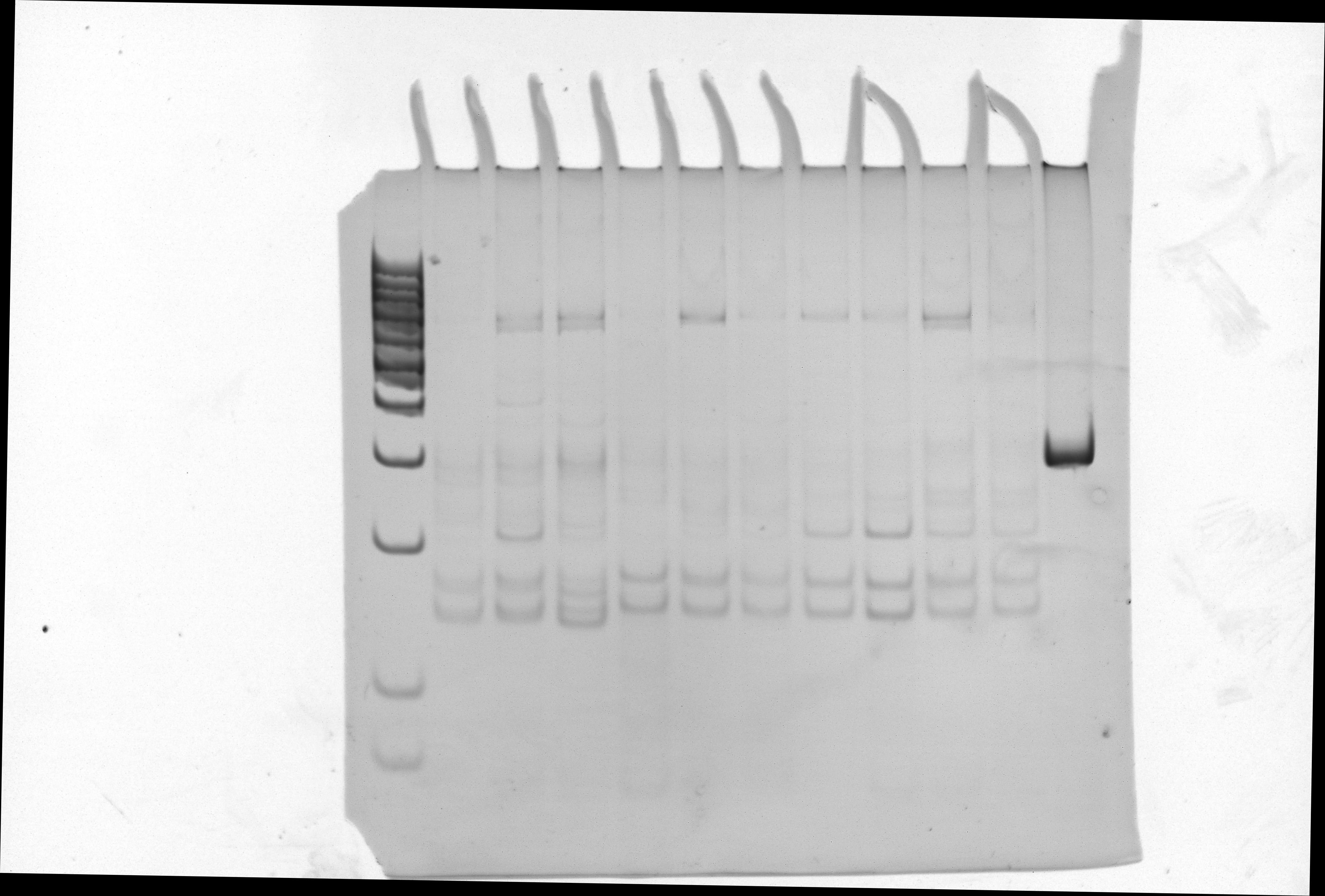

Supplement: Figure 1—source data 2. [file elife-89638-fig1-data2.zip › Figure1SourceData2_Fig1E_DNAGelsOriginal/20150916 patient-2610-2472-2477-2499-2516-D8S1752-2-invert.tif]

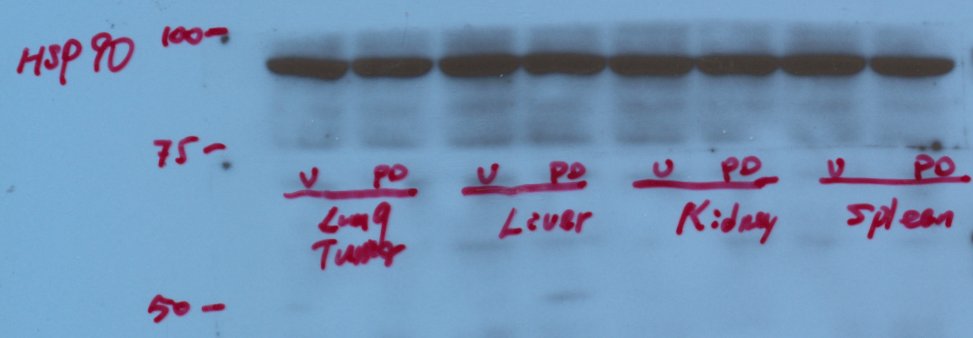

Supplement: Figure 3—source data 2. [file elife-89638-fig3-data2.zip › Figure3SourceData2_3BBlotsOriginal/TumorLiverKidenySpleen-VecPDLIM2 Hsp90_1.jpg]

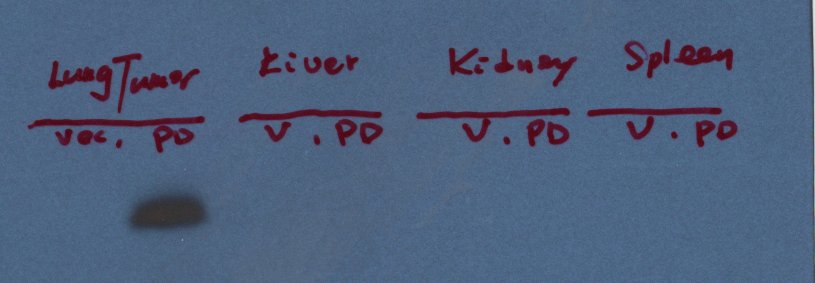

Supplement: Figure 3—source data 2. [file elife-89638-fig3-data2.zip › Figure3SourceData2_3BBlotsOriginal/TumorLiverKidenySpleen-VecPDLIM2 Myc.tif]
