# Supplementary material for: Improving PD-1 blockade plus chemotherapy for complete remission of lung cancer by nanoPDLIM2
Source: eLife. 2024 Dec 24;12:RP89638. doi: 10.7554/eLife.89638 (PMC11668523; doi:10.7554/eLife.89638)

Figure 1 --- Source Data 2
Related to Figure 1E

D8S1786-inverted

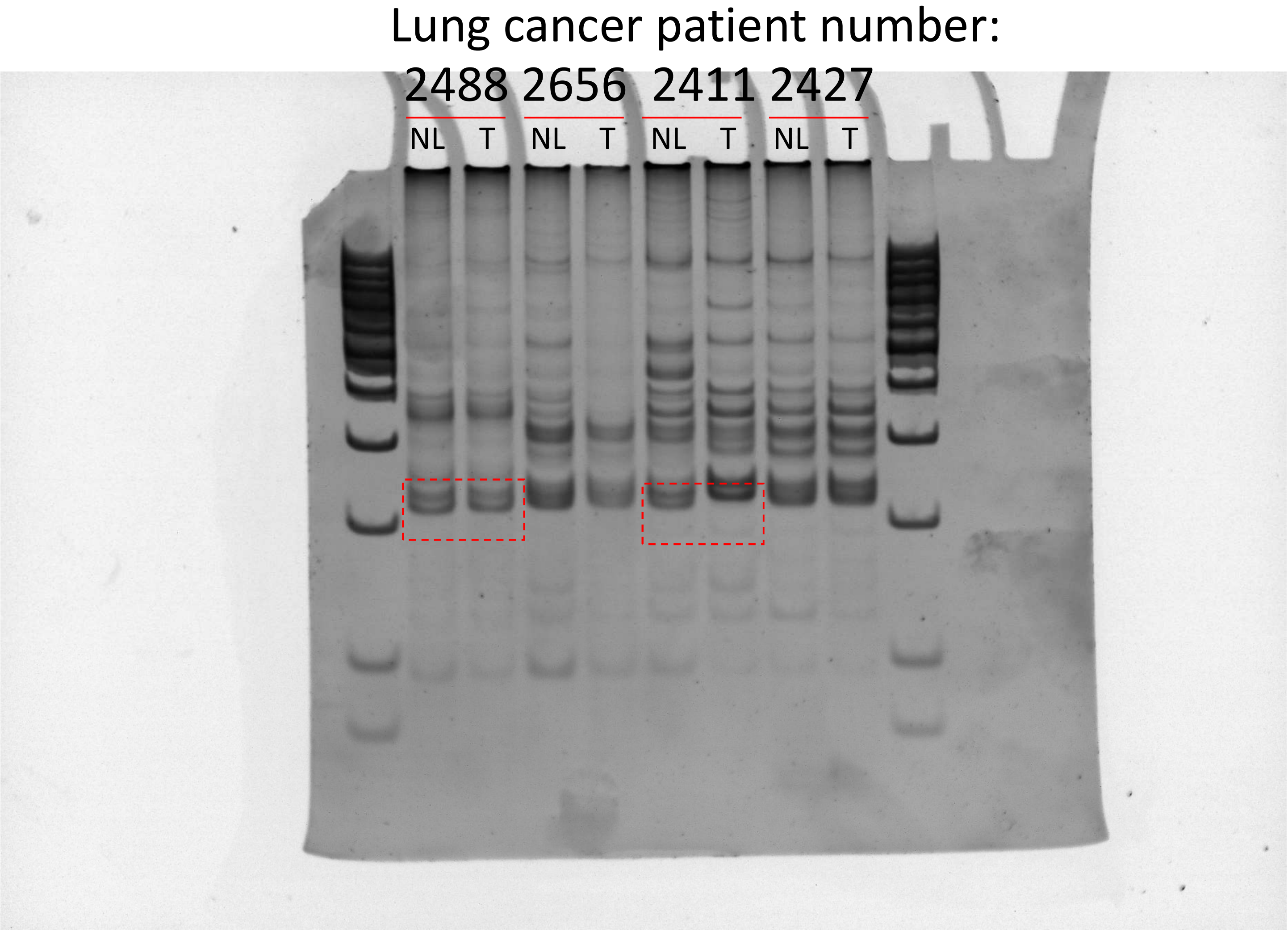

Figure 1 --- Source Data 2
Related to Figure 1E

D8S1752-inverted

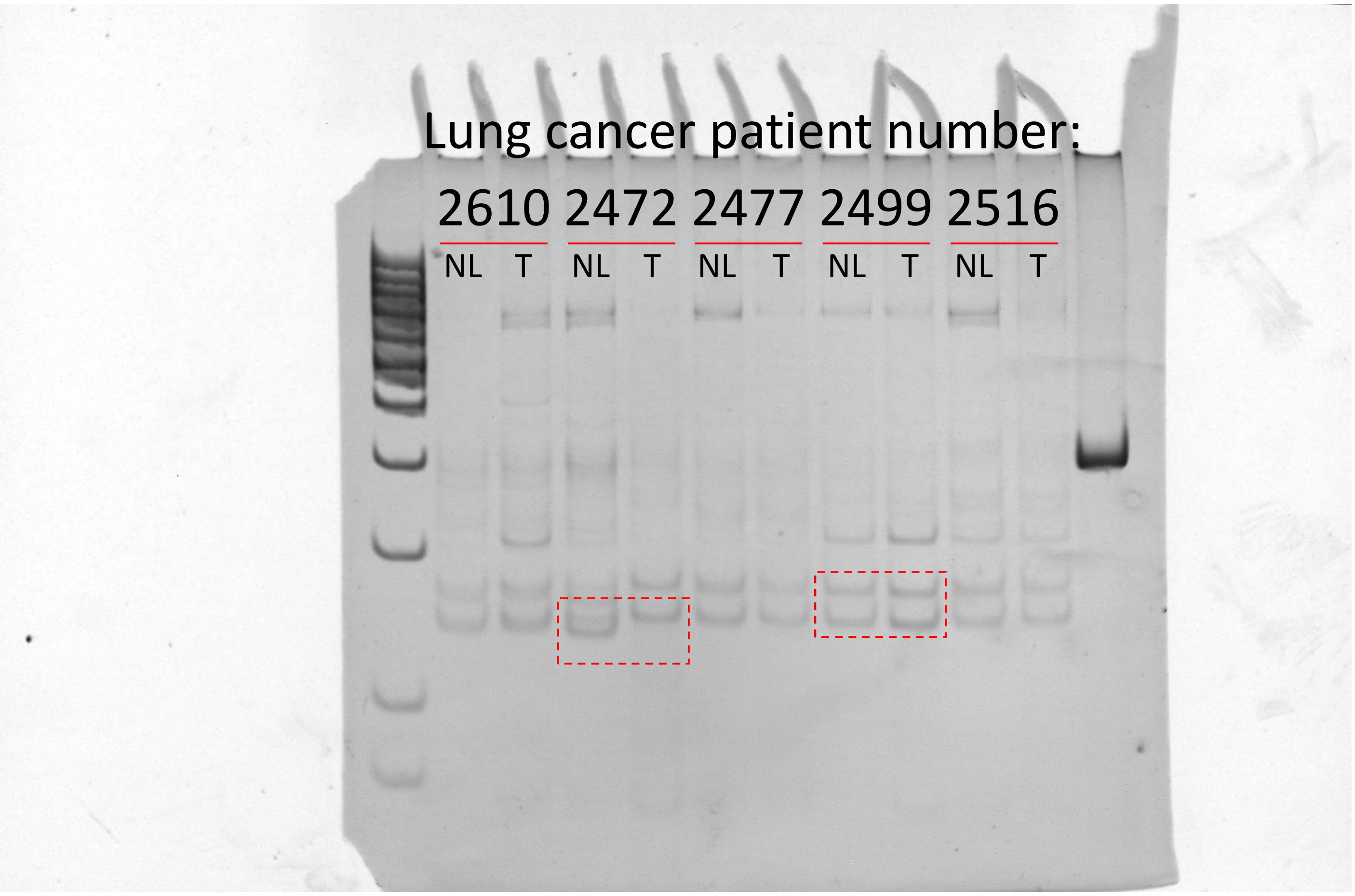

Supplement: Figure 1—source data 3. [file elife-89638-fig1-data3.zip › Figure1_SourceData3_Fig1E_DNAGel.pdf]
