# Supplementary material for: Improving PD-1 blockade plus chemotherapy for complete remission of lung cancer by nanoPDLIM2
Source: eLife. 2024 Dec 24;12:RP89638. doi: 10.7554/eLife.89638 (PMC11668523; doi:10.7554/eLife.89638)

Figure 3-Source Data 3

Western blot image shown in Figure 3B with the relevant bands clearly labelled

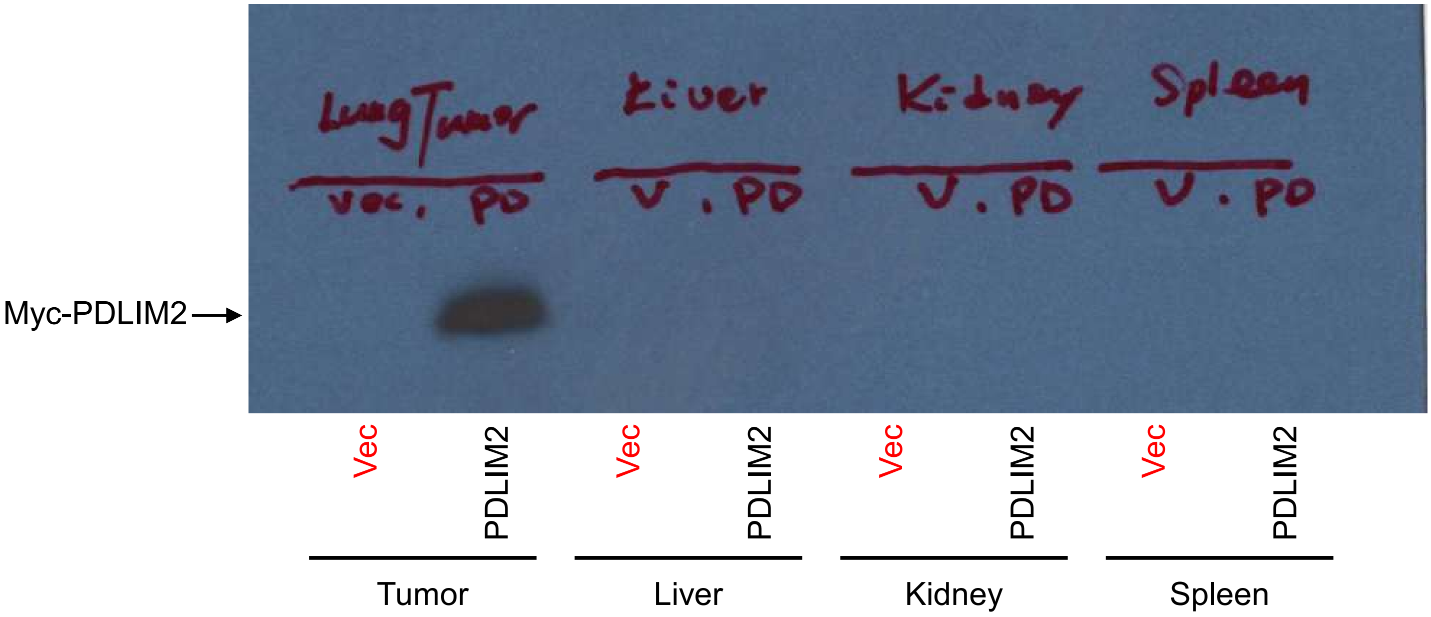

Figure 3-Source Data 3

Western blot image shown in Figure 3B with the relevant bands clearly labelled

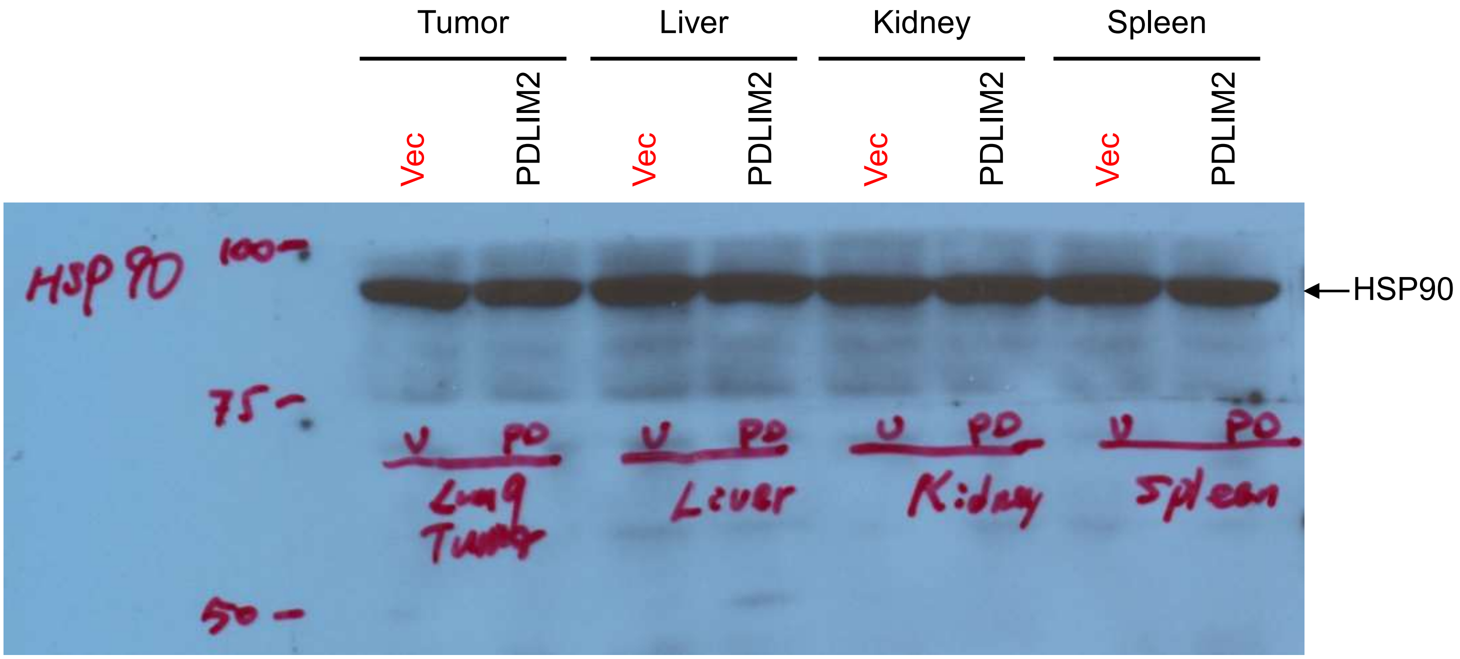

Supplement: Figure 3—source data 3. [file elife-89638-fig3-data3.zip › Figure3SourceData3_3BBlots.pdf]
