# Supplementary material for: Improving PD-1 blockade plus chemotherapy for complete remission of lung cancer by nanoPDLIM2
Source: eLife. 2024 Dec 24;12:RP89638. doi: 10.7554/eLife.89638 (PMC11668523; doi:10.7554/eLife.89638)
Supplement: Supplementary file 1. [file elife-89638-supp1.docx]

**Supplementary File 1a. Antibodies Used**

| **Antibody** | **Company** | **Cat. No.** | **Dose** | **Usage** |
| --- | --- | --- | --- | --- |
| Anti-PDLIM2 | Everest Biotech, Ramona, CA, USA | EB11878 | 1:400 | IHC |
| Anti-Cleaved Caspase 3 | Cell Signaling Technology, Danvers, MA, USA | 9661 | 1:200 | IHC |
| Anti-Bcl-xL | Cell Signaling Technology, Danvers, MA, USA | 2764 | 1:2400 | IHC |
| Anti-Cyclin D1 | Santa Cruz Biotechnology, Dallas, TX, USA | sc-450 | 1:200 | IHC |
| Anti-BrdU | Sigma-Aldrich, St. Louis, MO, USA | B2531 | 1:500 | IHC |
| Anti-RelA | Cell Signaling Technology, Danvers, MA, USA | 8242 | 1:500 | IHC |
| Anti-STAT3 | Cell Signaling Technology, Danvers, MA, USA | 4904 | 1:1000 | IHC |
| Anti-MDR1 | Cell Signaling Technology, Danvers, MA, USA | 13978 | 1:800 | IHC |
| Anti-CD4 | Abcam, Cambridge, MA, USA | ab183685 | 1:1000 | IHC |
| Anti-CD8 | Abcam, Cambridge, MA, USA | ab209775 | 1:1000 | IHC |
| Anti-PD-L1 | Cell Signaling Technology, Danvers, MA, USA | 64988 | 1:200 | IHC |
| Anti-MHC-I | Abcam, Cambridge, MA, USA | ab15681 | 1:200 | IHC |
| Anti-mouse IgG Biotinylated | Vector Laboratories, Burlingame, CA, USA | BMK-2202 | 1:200 | IHC |
| Goat anti-rat IgG Biotinylated | Vector Laboratories, Burlingame, CA, USA | BA9401 | 1:100 | IHC |
| Rabbit anti-goat IgG Biotinylated | Santa Cruz Biotechnology, Dallas, TX, USA | sc-2774 | 1:200 | IHC |
| Goat anti-rabbit IgG Biotinylated | Dako, Carpinteria, CA, USA | E0432 | 1:200 | IHC |
| Anti-Hsp90 | Santa Cruz Biotechnology, Dallas, TX, USA | sc-13119 | 1:1000 | WB |
| Anti-c-Myc | Santa Cruz Biotechnology, Dallas, TX, USA | sc-40 | 1:1000 | WB |
| Anti-CD16/CD32 | eBioscience, San Diego, CA, USA | 14-0161-85 | 1.0 μl per sample | FACS |
| Anti-CD45 FITC | Biolegend, San Diego, CA, USA | 103107 | 0.5 μl per sample | FACS |
| Anti-EpCAM PE | eBioscience, San Diego, CA, USA | 12-5791-82 | 0.625 μl per sample | FACS |
| Anti-MHC-I APC | eBioscience, San Diego, CA, USA | 17-5998-82 | 1.25 μl per sample | FACS |
| Anti-CD3 PE | eBioscience, San Diego, CA, USA | 12-0031-83 | 2.5 μl per sample | FACS |
| Anti-CD4 PE-Cy7 | eBioscience, San Diego, CA, USA | 25-0042-82 | 1.25 μl per sample | FACS |
| Anti-CD8a APC | eBioscience, San Diego, CA, USA | 17-0081-83 | 0.625 μl per sample | FACS |
| Anti-IFNγ FITC | eBioscience, San Diego, CA, USA | 11-7311-82 | 1 μl per sample | FACS |
| Anti-PD-1 | BioXcell, West Lebanon, NH, USA | BE0273 | 200 μg/mouse/time | *in vivo* blockade |

**Supplementary File 1b. Primers Used**

| **Gene** | **Species** | **Accession number** | **Forward (5' to 3')** | **Reverse (5' to 3')** | **Usage** |
| --- | --- | --- | --- | --- | --- |
| *D8S1786* | human |  | CGAAAGATTGAGACCCCAT | GTTTCCACACCGAAGCC | Microsatellite PCR |
| *D8S1752* | human |  | TCCTGGATCAGGCAGAAA | TCAGAGTTGGGTGAGCGA | Microsatellite PCR |
| *LINE-1* | human | M80343.1 | AAAGCCGCTCAACTACATGG | TGCTTTGAATGCGTCCCAGAG | RT-PCR |
| *PDLIM2* | human | NG_030435.1 | CAAAGCCAAGGACGCTGACC | GCTCTGGCGGATCTTGCTCT | RT-PCR |
| *pdlim2* | mouse | Mutant primer | GCTACCCGTGATATTGCTGAAGAG | | Genotyping  PCR |
|  |  | WT primer | GTCTTCTCTTCCTCAGGCATGGCG | |  |
|  |  | Common primer | GCCAACATTGCCCCAGAAGACTCC | |  |
| *Amp-R* | Plasmid |  | AACTTTATCCGCCTCCAT | GAAGCCATACCAAACGAC | Plasmid detection |
| *Lyz2* | mouse | NC_000076.6 | AGCAGGCATGCTTTCTCTAGTC | GCGATTAGCTGGAGCCATCA |  |
